# Impulse Control Disorders in Parkinson’s Disease: Crossroads between Neurology, Psychiatry and Neuroscience

**DOI:** 10.3233/BEN-129019

**Published:** 2013

**Authors:** Paulo Bugalho, Albino J. Oliveira-Maia

**Affiliations:** ^1^Department of Neurology, Hospital de Egas Moniz, Centro Hospitalar de Lisboa Ocidental, LisboaPortugal; ^2^Department of Neurology and CEDOC, Faculdade de Ciências Médicas, Universidade Nova de Lisboa, LisboaPortugal; ^3^Department of Psychiatry and Mental Health, Centro Hospitalar de Lisboa Ocidental, LisboaPortugal; ^4^Champalimaud Neuroscience Program, Instituto Gulbenkian de Ciência, Oeiras, Portugal and Champalimaud Centre for the Unknown, LisboaPortugal

**Keywords:** Parkinson’s disease, impulse control disorders, non-motor symptoms, frontostriatal circuits, dopamine

## Abstract

Non-motor symptoms contribute significantly to Parkinson’s disease (PD) related disability. Impulse control disorders (ICDs) have been recently added to the behavioural spectrum of PD-related non-motor symptoms. Such behaviours are characterized by an inappropriate drive to conduct repetitive behaviours that are usually socially inadequate or result in harmful consequences. Parkinson disease impulse control disorders (PD-ICDs) have raised significant interest in the scientific and medical community, not only because of their incapacitating nature, but also because they may represent a valid model of ICDs beyond PD and a means to study the physiology of drive, impulse control and compulsive actions in the normal brain. In this review, we discuss some unresolved issues regarding PD-ICDs, including the association with psychiatric co-morbidities such as obsessive-compulsive disorder and with dopamine related side effects, such as hallucinations and dyskinesias; the relationship with executive cognitive dysfunction; and the neural underpinnings of ICDs in PD. We also discuss the contribution of neuroscience studies based on animal-models towards a mechanistic explanation of the development of PD-ICDs, specifically regarding corticostriatal control of goal directed and habitual actions.

## 1. Introduction

Parkinson's disease (PD) is a neurodegenerative disorder characterized by loss of dopaminergic neurons in the substantia nigra. Clinical diagnosis depends on the presence of motor symptoms, namely tremor, rigidity, bradykinesia and gait disturbance [1]. However, PD patients also suffer from non-motor symptoms, such as cognitive dysfunction, behavioural disorders, sleep disorders, pain, dysautonomia and olfactory loss [2]. These symptoms have gained increasing attention from clinicians since they have been found to significantly influence the quality of life of PD patients, in some occasions even more than motor disturbances [3], and are generally related to poor prognosis [4]. Some non-motor symptoms appear early in the course of disease, sometimes preceding the appearance of motor dysfunction, making them valuable as predictive factors.

Psychiatric disorders, such as psychosis, depression and anxiety, are frequently found in PD [5]. The

ISSN 0953-4180/13/$27.50 © 2013 – IOS Press and the authors. All rights reserved spectrum of behavioural disorders in PD has recently been widened to include a group of conditions known as impulse control disorders (ICDs). These disorders are characterized by an inability to resist an inappropriate drive, usually of a hedonistic nature, which results in repetitive behaviours with harmful consequences. In PD, such behaviors can assume different syndromic presentations, which include pathological gambling; punding (compulsive engagement in meaningless hobby-like activities, including endless computer use, cleaning, gardening, dismantling electric house appliances and collecting worthless objects); hedonistic homeostatic behavioural disorder (the compulsive and excessive use of dopaminergic medication, irrespective of motor symptom improvement and usually at the expense of significant side-effects); hypersexuality; compulsive shopping; and binge eating. Impulse control disorders are not exceedingly common in PD, as compared to other psychiatric disorders: a global prevalence of 13.6% has been found in a large PD population, with gambling in 5.0%, compulsive sexual behaviour in 3.5%, compulsive buying in 5.7% and binge-eating disorders in 4.3% of patients [6]. They can, however, have great impact on the quality of life for PD patients [7] and those who care for them [8]. Interest in PD-ICDs also arose because PD constitutes a relevant biological model to study ICDs in functional psychiatric diseases [9].

The cause of ICDs in PD is still unknown. Some clinical features are overrepresented in PD patients with ICDs, such as longer disease duration, male sex, younger age [6,10–12], psychiatric co-morbidities (mainly depression), alcoholism, and a family history of ICDs or other addictive behaviours [6,11]. Personality characteristics, namely high scores in harm avoidance and neuroticism scales [13], as well as psychosocial factors leading to psychological distress [14], have also been proposed to contribute to the development of ICDs. However, dopaminergic treatment, particularly with dopamine agonists, is likely a key factor, as suggested by the association of dopamine agonist use and ICDs [6,15–17], the new-onset of ICDs in PD patients initiating dopamine agonist therapy [18] and the improvement of symptoms after reducing dopaminergic medication [19]. Furthermore, ICDs have also been described in patients receiving dopaminergic drugs for disorders other than PD, such as restless legs syndrome [20] and prolactinoma [21]. However, most patients under high doses of dopaminergic drugs do not develop ICDs [10,11], and these disorders have also been described in drug-naïve PD patients [22], which suggests that other factors must be at play. The effect of deep brain stimulation (DBS) treatment for PD further suggests that factors other than dopaminergic drugs could contribute for the development of ICDs in PD [23,24]. In theory, since DBS should allow for a reduction of the dopaminergic drug dose that is necessary for control of motor symptoms, it should have a beneficial effect in ICDs [24]. However, there is evidence that is not the case for all patients. Lim et al. [24], for instance, reported that in a group of 21 PD patients undergoing DBS, ICD did not improve or worsened after surgery in 71%. Some patients actually developed symptoms for the first time after DBS [24].

In this review we discuss some unresolved issues regarding ICDs in PD, namely the association with disorders of the obsessive-compulsive spectrum, with frontal cognitive deficits, and with dopamine related side-effects, such as psychosis and dyskinesia. Furthermore, we will review data from imaging studies regarding dopamine circuit dysfunction in ICDs and argue for a link between the clinical manifestations of ICDs and findings from animal research regarding the neural control of goal directed and habitual behaviours.

## 2. A connection between ICDs and Obsessive-Compulsive disorder?

Impulse control disorders share several features with Obsessive-Compulsive disorder (OCD), including the compulsion for repetitive actions and the inability to inhibit intrusive thoughts. One could thus consider ICDs in PD as belonging to the obsessive-compulsive spectrum, rather than an addiction related disorder as it is frequently considered in the literature [25]. Frontostriatal circuit dysfunction, a hallmark of PD [26], has been implicated in the pathophysiology of OCD [27]. Furthermore, obsessive-compulsive symptoms (OCS) can occur in patients with basal ganglia lesions [27], and are frequent in basal ganglia functional disorders, such as primary dystonia [28]. However, results from studies performed in PD have not led to definitive conclusions. Both Maia et al. [29] and Harbishettar et al. [30] did not find significant OCS in their PD patients. However, Alegret et al. [31] found OCS to be especially prevalent in particular PD groups, while Bugalho et al. [5] and Siri et al. [32] found a higher percentage of OCS in PD, using the same psychopathological screening scale. Studies addressing the relation between OCS and ICDs in PD are even scarcer. Antonioni and coworkers [22] tested this association and found that the ICD score was not significantly correlated to the OCS score, but rather with depression. Furthermore, the association of specific types of ICD with OCS has not been addressed. At a phenomenological level, different types of ICD could have different relations with OCS: while the repetitive nature of punding is reminiscent of OCD, hedonistic homeostatic behavioural disorder is similar to substance dependence and pathological gambling is akin to a behavioural addiction.

## 3. Cognitive dysfunction and frontal lobe deficits in PD-ICD patients

Frontostriatal circuits, mainly involving the orbitofrontal and dorsolateral prefrontal cortices, are essential for executive function, allowing for the selection of appropriate sets of actions and the concomitant inhibition of unwanted motor and behavioural programs. Frontal executive dysfunction, presumably caused by disturbance of frontostriatal loops, is the hallmark of cognitive dysfunction in PD [33,34]. Since impulse inhibition is one of the executive functions of the frontal lobe, one could hypothesize that frontal lobe dysfunction would underlie the presence of ICDs in PD patients. However, the evidence to support this hypothesis is inconsistent. Vitale and co-workers [35] found significant memory and executive deficits in PD patients with ICDs, when compared to a group of PD without ICD, matched for age and education. Another study found more specific differences, related only to executive type deficits [36]. In contrast, Siri et al. [37], found that PD patients suffering from pathological gambling (PG) performed better than other PD patients in several frontal lobe and non-frontal lobe type tasks. These authors hypothesized that preserved executive abilities could actually support the development of strategies to maintain gambling behaviour, such as lying to family members and other caregivers [37]. Still another study found an inverse relationship between memory tests (but not frontal function tests) and performance on the Iowa Gambling Task [38]. The inconsistencies among these results could stem from several factors, which include sampling bias (e.g., inclusion of different ICD types, or different proportions of each disorder) and the use of different neuropsychological batteries. Furthermore, in the Siri et al. study [37], younger age in the PG group and the presence of patients with dementia in the control group, could have contributed to the finding of better cognitive function in PD-PG patients. The remaining studies mentioned [35,36,38] controlled for age as well as gender and education.

Other variables, such as motor function, motor stage, dopaminergic treatment, and psychiatric comorbidity could also influence the association between frontal dysfunction and ICDs, possibly explaining the discrepancies between studies mentioned above. Another factor, which has not yet been investigated in the context of ICD, is the side of onset for motor symptoms. PD is an asymmetrical disorder, with motor symptoms typically starting on one side of the body, then progressing to axial structures and finally to the opposite side. Motor symptoms are usually more severe on the side of onset, and this difference persists until the very last stages of disease [39]. Asymmetry in motor symptoms is of course related to neuropathological asymmetry, with pathology occurring earlier on the brain hemisphere opposite to the side of motor onset [39,40]. Furthermore, motor and neuropathological asymmetry in PD could also be accompanied by asymmetry of non-motor symptoms, with left side parkinsonism associated to right hemisphere cognitive and behavioural symptoms and vice-versa [40]. In fact, there is evidence for an association of left-side onset PD with non-verbal executive and visuospatial deficits and with apathy; and of right-side PD with deficits on verbally mediated tasks and with anxiety [39,40]. Further research is necessary to understand if side of onset is also associated with ICDs in PD.

## 4. Association between ICDs and other dopamine-related side-effects

As mentioned previously, ICDs seem closely related to the use of dopaminergic drugs. Treatment with dopamine agonists has also been associated with other motor and non-motor symptoms, occurring mostly in the later stages of PD [41]. Motor fluctuations and peak-of-dose dyskinesia are well known complications of prolonged dopaminergic treatment [41]. Psychosis and visual hallucinations are also common in later stages of the disease, and are usually ameliorated by reducing the dose of dopaminergic drugs [41]. Some authors have thus proposed that dyskinesia, psychosis and ICDs could be different symptoms of the same pathophysiological continuum [41]. Moreover, some studies have found more severe dyskinesia in PD-ICD patients [42] and in PD patients suffering from punding [43]. However, a study testing a possible association between ICDs and psychosis had negative results [44] and other authors, using a general neuropsychiatric tool (the Neuropsychiatric Inventory), did not find significant differences between ICD and non-ICD patients on psychosis sub-scores [37]. Thus, the exact relationship between treatment-induced dyskinesia, psychosis and ICDs remains to be determined. Further research is necessary to clarify if such a relationship exists [41].

## 5. Neural underpinnings of ICDs in PD

The fact that ICDs may occur in association with PD, a neurodegenerative disease with a well-defined neuromodulatory imbalance [45], has led to intense interest in these conditions, as an approach to better understand ICDs beyond PD [46]. Given the nature of PD, the contribution of dopaminergic dysfunction has been a frequent consideration. There is evidence that PD patients are biased towards learning from negative outcomes (avoidance) rather than positive outcomes (approach), and that such bias is reversed by dopaminergic medication [47], particularly in PD patients with ICDs [46]. Furthermore, [$^{11}$C]-raclopride positron emission tomography (PET) studies of PD patients have shown enhanced dopamine release in the ventral striatum of patients with pathological gambling while they were gambling [48] (Fig. 1A), and in patients with hedonistic homeostatic behavioural disorder, in response to L-dopa administration [49] (Fig. 1B). Functional magnetic resonance imaging (fMRI) studies have also indicated reduced neural activity in the striatum of PD patients with ICDs [50,51], but this has not been universally replicated [52]. These findings are consistent with changes in dopamine homeostasis [53] and neural activation [54] found in non-PD patients with chemical or behavioural addictions.

Dopamine reuptake has generally been less explored in the context of ICDs, particularly in PD patients [55]. A recent publication describes that PD patients with pathological gambling have reduced density of dopamine transporter (DAT) in the ventral striatum, as measured by [$^{123}$I]FP-CIT single photon emission tomography (SPECT) scans (DaTscan) [55]. This finding could be due to ventral striatal DA terminal loss, which seems unlikely given the reports of enhanced dopamine release in the ventral striatum of PD-ICD patients [48,49], or lower membrane expression determined functionally or genetically [55]. DAT gene variants have been consistently demonstrated in non-PD patients with ICDs and addiction [56–60], but a single study with PD-ICD patients found no association between the presence of ICDs and one particular DAT polymorphism [61].

As described above, most published research regarding changes in dopamine homeostasis in PD-ICD patients have targeted specifically those patients with pathological gambling or related chemical or behavioural addiction-like disorders. To date, there is very limited work to test for the presence of such changes in patients suffering from punding. However, as described above, at a phenomenological level punding seems to differ relative to other ICDs in PD. Given the relationship between punding and dyskinesia [43], and since the development of dyskinesia is associated to dopaminergic changes in dorsal areas of the striatum [62] (Fig. 1C), it is thus possible that, contrary to the remaining ICDs, punding may be more closely associated to changes in dopamine homeostasis localized to the dorsal striatum.

## 6. Using neuroscience to understand ICDs

The several conditions of the PD-ICD spectrum recapitulate the characteristics of several diseases of a putative impulsive-compulsive spectrum, which includes OCD, body dysmorphic disorder, Tourette's syndrome, trichotillomania, attention deficit hyperactivity disorder, pathological gambling, and substance addiction [63]. In these disorders, compulsivity and impulsivity have been deconstructed into several distinct neurocognitive mechanisms, including motor impulsivity, measured by the stop signal reaction time [64,65]; decision-making impulsivity, measured by gambling tasks such as the Cambridge gambling task [66] and Iowa gambling task [67]; reflection impulsivity, measured by information sampling tasks, such as the reflection task [68]; and cognitive inflexibility, measured by reversal learning [69] and attentional set-shifting tasks [70].

In an attempt to clarify the mechanisms that underlie normal neuropsychological function in these domains, as well as the dysfunction associated with disorders in the impulsive-compulsive spectrum, adaptations of many of the tests and tasks described above have been applied to animal models [71–77]. This has enabled invasive experimental approaches, such as lesion studies and local pharmacological manipulation, resulting in a more extensive description of neuroanatomical and neurochemical factors with possible translational relevance for these disorders, as well as the development of models attempting to explain the neurobiological con-

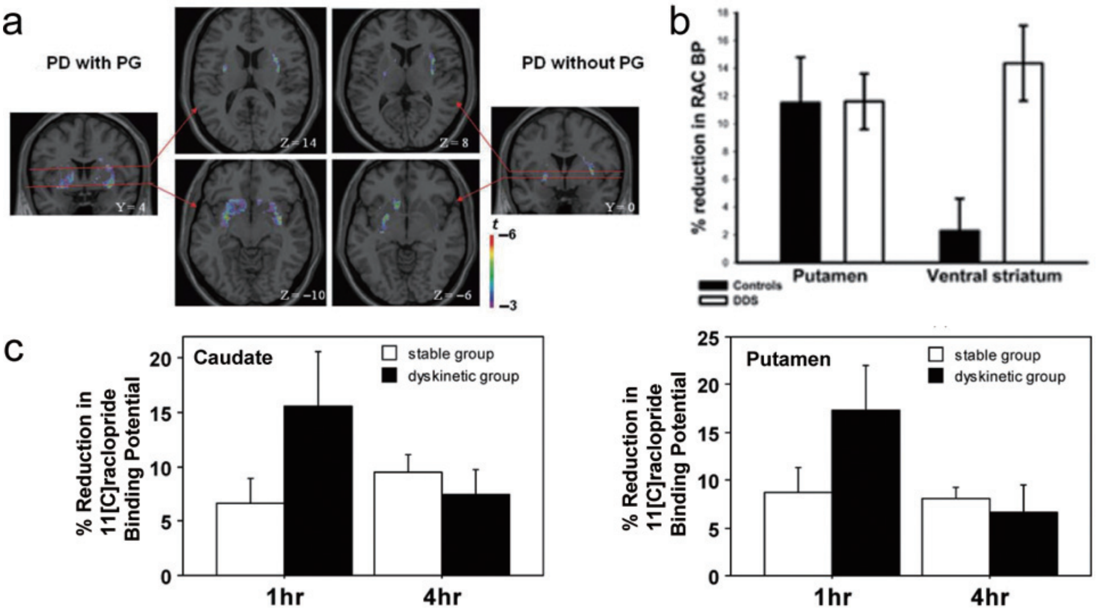

Fig. 1. [11][C]raclopride PET can be used to estimate dopamine release, which is considered to correlate with reduction of [11][C]raclopride binding potential, in response to a pharmacological, behavioral or other challenge. (**a**) This approach has been used to compare responses to gambling in PD patients with pathological gambling (PD with PG – 3 panels on the left side) vs. PD patients without pathological gambling (PD without PG – 3 panels on the right side). These panels show coronal and axial sections of the statistical parametric map of the change in [11][C]raclopride binding potential during gambling, overlaid on the average MRI in stereotaxic space. When compared to PD patients without PG, PD patients with PG displayed significantly more dopamine release in ventral areas of the striatum (bottom panels) in response to gambling, but not in dorsal areas (upper panels) [48]. (**b**) A similar approach has been used comparing PD patients with excessive use of dopamine medication (DDS – dopamine dysregulation syndrome) and other PD patients (controls). More dopamine release was found in the ventral striatum of DDS patients, in response to an L-dopa challenge [49]. (**c**) [11][C]raclopride PET after an L-dopa challenge has also been used to compare PD patients with and without dyskinesia, showing increased release of dopamine in more dorsal areas of the striatum in the dyskinetic group [62]. Adapted from [48, 49,62] with permissions.

trol of impulsive and compulsive behaviour [63]. Irrespective of all the advances in understanding impulsive and compulsive behaviour, there are still many unresolved issues and missing links, not the least of which being the role of dopamine. One predominant hypothesis proposes expression of dopamine type 2-like receptors in the ventral striatum as a marker for trait impulsivity [78], conferring susceptibility for psychostimulant drug addiction [78,79], a finding which has also been reported in humans and for other non-stimulant drugs [80]. In any case, understanding further the development of ICDs in PD could prove to be a relevant contribution towards explaining the role of dopaminergic and striatal function for impulsive-compulsive spectrum disorders.

A recent proposal to conceptualize the role of dopaminergic and striatal function for impulsive-compulsive spectrum disorders, and specifically for PD-ICDs, involves considering the participation of the basal ganglia in the control of learned actions [41]. Learned actions have been categorized by behavioural science, and more recently neuroscience, according to their sensitivity to devaluation [81–83] and the contingency between action and outcome [84–86] (Fig. 2). Goal directed actions are governed by the associative structure between action and outcome, and their performance will thus be reduced by manipulations that reduce the value of the outcome (Fig. 2B) or eliminate the contingency between action and outcome (Fig. 2C). Habitual actions, on the other hand, are governed by a stimulus-response relationship, and will be sustained even if the outcome is no longer valuable (outcome devaluation, Fig. 2B) or if the action no longer leads to the outcome (contingency degradation, Fig. 2C). Experiments conducted in rats have shown that several manipulations, including overtraining on a particular schedule of reinforcement, can result in a habitual pattern of behaviour [81,82], and the same finding has been reported in humans [87]. This property of learned actions could be relevant for disorders such as ICDs and addiction, where previously reinforced behaviour is sustained, irrespective of current negative consequences [41].

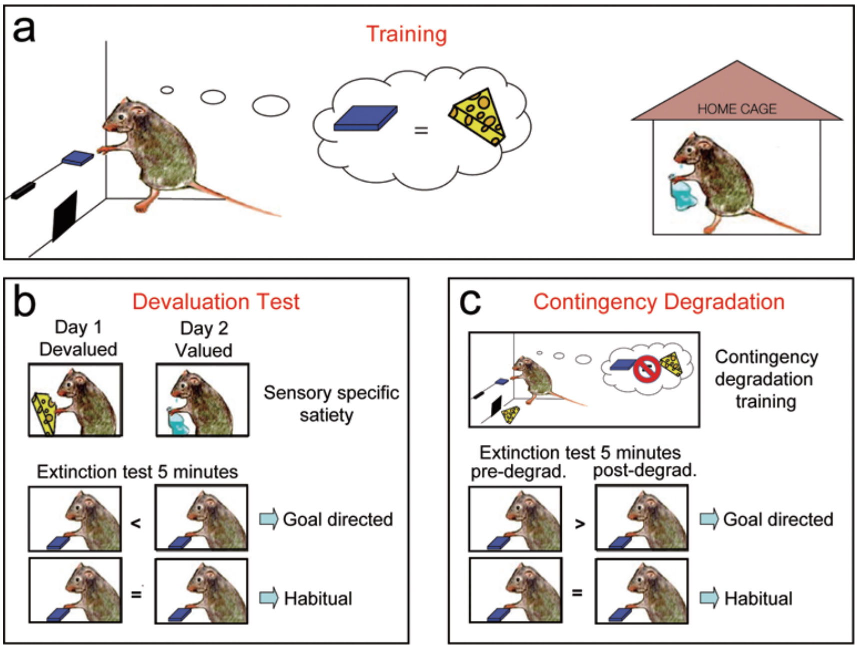

Fig. 2. This figure represents one of the several experimental protocols that can be applied to rodents to test the goal directed vs. habitual nature of learned incentive actions. (**a**) Animals are first trained with one of two reinforcers. The depiction here is of cheese being delivered in the operant box, contingent upon lever pressing, with the other reinforcer, a sucrose solution, delivered freely in the home cage (note that the types of reinforcers described are for illustrative purposes only). (**b**) Devaluation testing is typically performed in two days, using a protocol of sensory specific satiety, after a period of daily training as in (a). On either of these two days, prior to being introduced in the operant chamber, the animals are given the reinforcer previously obtained by lever pressing, in this case cheese (devalued condition, day 1 in the figure), or, on an alternate day, the reinforcer previously obtained freely in the home cage, in this case sucrose solution (valued condition, day 2 in the figure). Immediately after these feeding sessions, the animals are placed in the operant box for a 5 minute test conducted in extinction, i.e., they can press the lever but no reinforcers are earned. The behaviour is considered goal directed if the animals press more in the valued than the devalued condition, and habitual if number of presses are not different across the two days. (**c**) Contingency degradation is another protocol to test if behaviour is goal directed or habitual. Here, after training as in (a), animals are re-trained in the operant box but delivery of the reinforcer previously obtained by lever pressing, in this case cheese, is no longer contingent upon lever pressing, i.e., cheese is delivered freely, irrespective of lever pressing. After the contingency degradation training, a 5 minute extinction test is conducted and the number of lever presses is compared with those obtained in an extinction test conducted prior to extinction, or in another group of animals that were nor exposed to contingency degradation training. The behaviour is considered goal directed if the animals press more in the non- or pre-degraded than the post-degraded condition, and habitual if the number of presses is not different across the two conditions. Adapted from [104] with permissions.

Differential neural substrates for goal directed and habitual action have been extensively demonstrated [88–95] (Fig. 3). In rats, bilateral lesions of the dorsolateral striatum [91,96], as well as lesions of the nigrostriatal dopaminergic pathway that innervates the dorsolateral striatum [97], lead to resistance to the development of habits. Conversely, the dorsomedial striatum is involved in goal directed behaviour [92, 93], whereas the ventral striatum mediates how reward-related sensory cues influence the performance of learned incentive actions [98]. Deficits in reinforcement learning have been shown for PD patients [47], and such deficits are reverted by dopaminergic medication, particularly in PD-ICD patients [46]. However, in these patients, more specific aspects of reinforcement learning, such as sensitivity to outcome devaluation and contingency degradation, remain to be tested.

## 7. Discussion

Much remains to be investigated in the field of ICDs and their relation with PD. We propose that research on the physiopathology of PD-ICDs would gain from a more complete understanding of the connection with other non-motor symptoms attributed to frontostriatal dysfunction, such as frontal cognitive dysfunction and OCD. Furthermore, and in accordance with previous research, we have underscored the importance of investigating the influence of dopamine dysfunction, either through the use of imaging techniques to directly probe dopaminergic circuit function, or by assessing the relationship between ICDs and other symptoms of dopaminergic-drug-related side effects, typical of advanced stage PD. As such, the heterogeneity of ICD

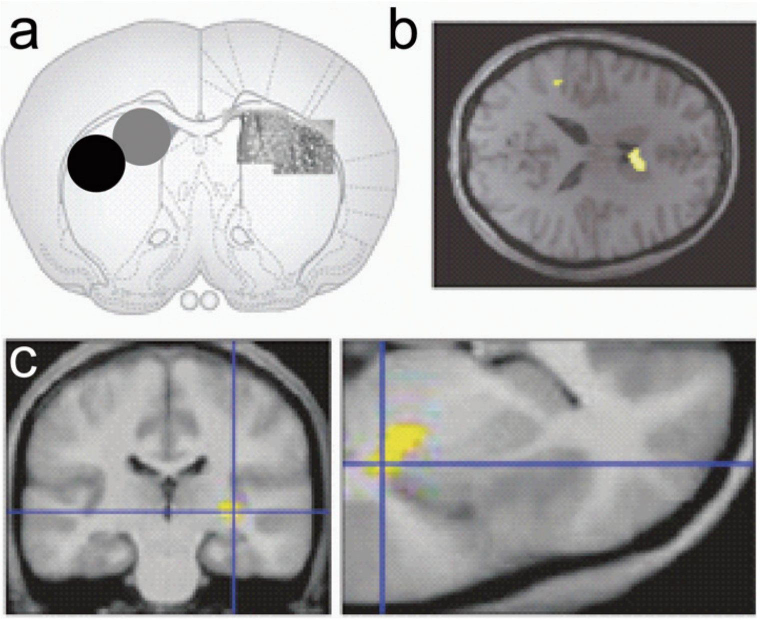

Fig. 3. Here we show evidence for homologies between humans and rodents in striatal control of goal directed and habitual actions. (**a**) This panel shows a diagram representing a coronal slice of the rat brain, at a level where the striatum is depicted. On the right hemisphere, photo-micrographs of NMDA-induced cell-body lesions of dorsomedial and dorsolateral striatum have been superimposed on the diagram while on the left hemisphere the approximate region of lesion-induced damage is illustrated using gray and black circles, respectively. In this species, bilateral lesions of the dorsomedial striatum were found to impair goal-directed learning [93] while lesions of the dorsolateral striatum impair habit learning [91]. (**b**) This panel shows fMRI data from humans performing an incentive task. Activity in the anterior dorsomedial striatum was sensitive to instrumental contingency, i.e., had response properties consistent with the goal directed system [105]. (**c**) Region of posterior lateral striatum (posterior putamen) exhibiting increasing response with training, across a training protocol that promotes development of habitual behaviour [87]. Adapted from [94] with permission.

syndromes in PD should be taken in account, since they may represent different pathophysiological entities and thus bear different relationships with other clinical and neurobiological features.

A more complete understanding of PD-ICDs, and particularly of the underlying neurobiology, could lead the way to novel therapeutic approaches, possibly even applicable to other disorders of the impulsive-compulsive spectrum. The use of neuromodulatory approaches, already in clinical use for the treatment of motor symptoms in PD [23,24], could be an interesting alternative. Promising findings have been reported for non-invasive modulation of prefrontal cortex activity to modify risk-taking behaviour in human volunteers [99,100], even those of older ages [101], during a gambling task. Furthermore, transcranial direct current stimulation of the dorsolateral prefrontal cortex has been shown to transiently reduce alcohol craving in patients with alcohol dependence [102], while repetitive transcranial magnetic stimulation of the same brain area transiently reduces cocaine craving in patients with cocaine dependence [103]. In this context, PD-ICDs could represent a valuable model to understand basic mechanisms of volition and impulse control, as well as other disorders of the impulsive-compulsive spectrum, possibly stimulating further exploration of neuromodulatory approaches in the treatment of these disorders, in a fruitful interaction between 'bench and bedside' [46], with potential benefits for both neuroscience and clinical neuropsychiatry.

## Acknowledgments

We would like to thank Rui M. Costa, Eduardo Dias-Ferreira and Joaquim Alves da Silva for valuable discussion and Ana B. Fernandes for review of the manuscript. Preparation of this manuscript was supported in part by a Junior Research and Career Development Award from the Harvard Medical School – Portugal Program, a grant from the BIAL Foundation and a fellowship from the AXA Research Fund to AJO-M.
